# The Effects of Reduced Glutathione on Growth Performance, Intestinal Inflammation, and Gut Microbiota in Immune-Stressed Broiler Chickens

**DOI:** 10.3390/ani16020178

**Published:** 2026-01-07

**Authors:** Xin-Qi Wang, Tao Zhang, Ying-Kun Liu, Hao-Jia Li, Kabelo Anthony Makatjane, Zhen Lai, Jian-Xin Bi, Hai-Zhu Zhou, Wei Guo

**Affiliations:** 1 College of Animal Science and Technology, Jilin Agricultural University, Changchun 130118, China; 18943857722@163.com (X.-Q.W.); 18188114727@163.com (Y.-K.L.); 18932783018@163.com (H.-J.L.); anthonymakatjane@gmail.com (K.A.M.); 13147728730@163.com (Z.L.); 13251763422@163.com (J.-X.B.); 2 Jilin Provincial Science and Technology Innovation Platform Management Center, Changchun 130012, China; anbigboy@126.com; 3 Jilin Provincial Livestock Station, Changchun 130000, China

**Keywords:** reduced glutathione, white feather broiler, immune stress, intestinal flora

## Abstract

In intensive farming systems, broilers are exposed to various stressors that trigger immune stress. Reduced glutathione is known to play a crucial role in modulating immune function. This study investigated the effects of different doses of reduced glutathione on the growth performance, intestinal immune function, and gut microbiota of broilers under immune stress. One-day-old broilers were randomly assigned to five groups: group K (control), group L (lipopolysaccharide), and three glutathione supplementation groups, Y1 (50 mg/kg), Y2 (100 mg/kg), and Y3 (200 mg/kg). Each group had four replicates, with 11 broilers per replicate. On days 16, 18, and 20, broilers in groups L and Y1-Y3 received intraperitoneal injections of 0.5 mg/kg of lipopolysaccharide, while group K received an equal volume of saline. On day 16, the average daily weight gain and final body weight of group Y3 were significantly higher than those of groups K and L, whereas on day 21, the average daily weight gain of group Y3 was still significantly higher than that of group L. On day 21 broilers were slaughtered, and samples were collected. In the jejunal mucosa, group Y3 showed significantly reduced levels of IL-2, IL-4, IL-1β, and TNF-α compared to group L. Additionally, group Y3 exhibited reduced relative expression levels of *NF-κB*, *TLR4*, *IFN-γ*, *IL-1β*, *IL-6*, *IL-4*, *TNF-α*, and *IL-2* mRNA; a decreased abundance of *Enterococcus*, *Proteobacteria*, and *Actinobacteria*; and a restored abundance of *Ligilactobacillus*, *Turicibacter*, and *Firmicutes* to normal levels. These findings suggest that 200 mg/kg of reduced glutathione is the optimal dose for improving the gut microbiota composition and mitigating gut damage caused by immune stress by inhibiting the *TLR4/NF-κB* signaling pathway.

## Simple Summary

In modern animal production, factors such as environmental stressors, dietary changes, pathogenic microorganisms, and toxins may trigger immune stress reactions. These reactions can severely affect animal growth and development, damage gastrointestinal integrity, and, in extreme cases, even lead to mortality. Reduced glutathione, as an endogenous antioxidant, has detoxification, antioxidant, and immune functions in stressed broiler chickens. This study involved a 21-day feeding experiment to investigate whether GSH could mitigate the negative impacts of lipopolysaccharide LPS-induced immune stress in broiler chickens. Our results demonstrate that dietary supplementation with 200 mg/kg of GSH alleviates LPS-mediated inflammatory damage and increases the levels of beneficial bacteria in the gut microbiota by inhibiting the inflammatory cytokine signaling pathway TLR4/NF-κB.

## 1. Introduction

In the commercial poultry industry, broiler chickens are often raised in high-density environments. Such conditions often trigger stress reactions due to environmental or pathogenic microorganisms, which subsequently lead to damage to the intestines of broiler chickens. The very same intestines serve as both the primary organ for digestion and nutrient absorption and the largest immune organ in the body [1]. The colonizing gut microbiota produce microbial metabolites that influence local or systemic immune or nutritional functions of the body [2]. Under ideal or homeostatic conditions, the diversity and quantity of the gut microbiota remain relatively balanced [3]. However, during immune stress, the gut structure is damaged, and immune cells release pro-inflammatory cytokines [4]. These cytokines bind to cell surface receptors to activate the NF-κB signaling pathway, thereby regulating the transcription and expression of inflammation-related genes. These changes typically lead to a decrease in the diversity of the gut microbiota, changes in body metabolites, and a decrease in the nutrient absorption rate, ultimately affecting growth performance. The gut microbiota is essential for maintaining intestinal integrity and barrier function, regulating immune function, and protecting the body from pathogen invasion [5].

Lipopolysaccharide (LPS), the main pathogenic component of Gram-negative bacteria cell walls, is also known as a cellular endotoxin. As a potent stressor, it is widely used in the establishment of immune stress models in poultry [6,7,8]. LPSs activate the immune system of poultry upon bacterial lysis or artificial destruction. Once released LPS interacts with host immune cells, ultimately activating the TLR4/NF-κB signaling pathway through a cascade reaction of the cell membrane and intracellular pathways [9,10]. Studies demonstrated that intraperitoneal injections of LPS induce inflammation, growth inhibition, and even mortality [11].

Glutathione is a major non-enzymatic antioxidant widely present in mammalian cells, consisting of a tripeptide composed of glutamic acid, cysteine, and glycine [12,13]. In living organisms, glutathione exists dynamically in two forms: reduced glutathione (GSH) and oxidized glutathione (GSSG). The ratio of GSH to GSSG is a key indicator for evaluating the cellular oxidative stress status [14], and the two forms are interconverted through redox reactions [15] to maintain cellular redox homeostasis. The imbalances in this steady state can lead to various pathological processes, such as immune disorders, metabolic diseases, and inflammation [16,17]. Research indicates that the exogenous supplementation of glutathione or its precursors can effectively eliminate free radicals and enhance the body’s antioxidant and immune capabilities, thereby improving animals’ growth performance and promoting their growth and development [18,19,20]. Consequently, glutathione is considered a green and safe additive with broad application prospects as a potential alternative to antibiotics.

At present, while glutathione is widely used in the research on aquatic organisms, there is little research on its use as a feed additive for poultry. This study utilizes an LPS-induced stress model to explore the effects of reduced glutathione on the growth performance, immune function, and gut microbiota in broiler chickens. By determining the optimal dose of reduced glutathione, this research aims to provide a reference for its use as a feed additive in the poultry industry.

## 2. Materials and Methods

All animal experiments were approved by the Ethics Review Committee of Jilin Agricultural University. All animal experiments were conducted at the Animal Experiment Base of Jilin Agricultural University.

### 2.1. Experimental Design and Animal Feeding Management

A total of 220 1-day-old AA broiler chickens were randomly divided into 5 groups, with 4 replicates in each group and 11 chickens in each replicate. The experimental period lasted 21 days. The birds were fed a basal diet supplemented with reduced glutathione (GSH) at different concentrations for 21 consecutive days: 0 mg/kg (K group), 0 mg/kg (L group), 50 mg/kg (Y1 group), 100 mg/kg (Y2 group), and 200 mg/kg (Y3 group). The composition of the basal diet was the same for all groups (Table 1). On days 16, 18, and 20, the L, Y1, Y2, and Y3 groups were intraperitoneally injected with lipopolysaccharide (LPS, 500 μg/kg BW), whereas the K group received the same volume of physiological saline. The administration route and dosage of LPS were based on the study by Li et al. [21]. The experimental animals were kept in an environment with 50–70% humidity. The room temperature was controlled at 32–34 °C for the first week and was subsequently lowered by 1 °C every two days until it reached 21 °C. A combination of natural and artificial light was used. The lighting schedule consisted of 23 h of light and 1 h of darkness, featuring strong-intensity light irradiation for the first week, followed by 18 h of light and 6 h of darkness, featuring weak light irradiation. The distribution of the light intensity was uniform. Afterwards, the birds were exposed to natural light. Water and feed were provided ad libitum. *Escherichia coli* (serotype O55: B5) lipopolysaccharide (LPS, L2880) with a purity of ≥99% and glutathione (GSH, 23030701) was purchased from Sigma Aldrich (St. Louis, MO, USA). 

### 2.2. Sample Collection

The broiler chickens were euthanized by cervical dislocation. The middle section of the jejunum was dissected using sterile surgical scissors and rinsed with physiological saline. The mucosal samples from the middle section of the jejunum were scraped using sterile glass slides and stored in a −80 °C refrigerator for subsequent measurement.

### 2.3. Growth Performance

The residual feed was weighed and recorded each morning before feeding to calculate the daily feed intake. The experimental broiler chickens were weighed after fasting at 1, 16, and 21 days of age. The average daily weight gain (ADG), average daily feed intake (ADFI), and feed-to-gain ratio (F/G) were calculated for a period of 1 to 21 days.

### 2.4. Intestinal Inflammation

The middle segment of the jejunal mucosa was harvested for biochemical analysis. The levels of secreted immunoglobulin A (sIgA, μg/mL), immunoglobulin G (IgG, μg/mL), and immunoglobulin M (IgM, μg/mL) alongside pro-inflammatory cytokines, including interleukin-2 (IL-2, pg/mL), interleukin-4 (IL-4, pg/mL), interleukin-6 (IL-6, pg/mL), interleukin-1 β (IL-1β, pg/mL), and tumor necrosis factor-α (TNF-α, pg/mL), were determined using an enzyme-linked immunosorbent assay (ELISA kit). All procedures were performed strictly according to the manufacturer’s protocols (Shanghai Enzyme Linked Biotechnology Co., Ltd., Shanghai, China).

### 2.5. Expression Levels of TLR4/NF-κB Signaling Pathway-Related Factors

The total RNA was extracted from 0.5 g of jejunal mucosal tissue using a Trizol reagent (Invitrogen™, Carlsbad, CA, USA). The concentration and purity of the extracted RNA were quantified using an ultramicrospectrophotometer (Nanodrop 2000, Thermo Fisher, Waltham, MA, USA); only samples with an OD260/OD280 ratio between 1.8 and 2.1 were used for further analysis. Subsequently, the total RNA of each sample was reverse transcribed into cDNA according to the Takara kit (Bao Bioengineering Co., Ltd., Dalian, Liaoning Province, China) instructions. A real-time fluorescence quantitative PCR (RT-qPCR) was performed in a 20 μL solution using SYBR Green I (Bao Bioengineering Co., Ltd., Dalian, Liaoning Province, China) as the fluorescent dye. The specific chicken primers *TNF-α*, *IL-1β*, *IL-2*, *IL-6*, *IFN-γ*, and *NF-κB* were in the Premier 6 software based on sequences in the NCBI gene database. β—actin served as the internal reference gene. The relative expression levels of each gene were calculated using the 2^−∆∆Ct^ method. All primers were synthesized by Shanghai Biotechnology Co., Ltd. in China, and their sequences are shown in Table 2.

### 2.6. Analysis of Gut Microbiota

Based on the optimal concentration of GSH determined in the previous experiment, the total DNA of the jejunum contents was extracted from the control group and LPS-stressed group using a commercial DNA extraction kit: the DNA purity and concentration were detected by 1% agarose gel electrophoresis. The V4 hypervariable region of the bacterial 16 S rRNA gene was amplified using the universal primers 515F (CAGCMGCCGCGCGTGTGTAA) and 806R (TACHVGGGTWTCTAAT). The PCR products were validated using 2% agarose gel electrophoresis, and the qualified products were purified by magnetic beads and quantified via an enzyme-linked immunosorbent assay. After full mixing, the Equimolar amounts of purified PCR products were pooled and recovered from 2% agarose gels. Libraries were constructed and quantified using Qubit and Q-PCR before being sequenced on the NovaSeq6000 (PE250) platform (Illumina Inc., San Diego, CA, USA). The raw data was demultiplexed based on the unique barcode and primer sequences. The initial quality control step and sequence assembly were performed using FLASH (Version 1.2.11, http://ccb.jhu.edu/software/FLASH/, accessed on 5 January 2026) (Magoc T. et al. 2011 [22]) to generate raw tags. Subsequent processing included the use of the Cutadapt software v4.2 to remove reverse primer sequences. After the above processing, the obtained tags were further processed to remove the chimeric sequence. The tag sequence was annotated with the species annotation database (Silva database) https://www.arbsilva.de/ (accessed on 5 January 2026) for 16S/18S, Unite database https://unite.ut.ee/ (accessed on 5 January 2026). For ITS, the chimeric sequences were compared and detected, and the chimeric sequences were ultimately removed to obtain the final effective tags (Edgar R.C. et al., 2011 [23]). The DADA2 module or deblur in the QIIME2 (Version QIIME2-202202) software was employed to denoise the sequences (default to DADA2), yielding high-resolution Amplitude Sequence Variants (ASVs) and a feature table. Sequences were aligned against the Silva138.1 database (for 16S) using QIIME2. After the OTU clustering analysis and species taxonomy analysis on samples with 97% similarity, a multiple diversity index analysis was performed based on OTUs. The alpha and Beta indices were calculated using QIIME2 software. A Principal Coordinate Analysis (PCoA) was conducted and plotted using the ade4 and ggplot2 packages in R software (version 4.0.3). Species abundance statistics were based on the abundance of each sample at different classification levels (phylum, genus), and a distribution histogram of the relative abundance was drawn using SVG functions.

### 2.7. Data Analysis

The raw data was processed using Excel. GraphPad Prism 8.0 was used for plotting, and SPSS 26.0 software was used for the Shapiro–Wilk test, the one-way ANOVA, and Duncan’s multiple comparisons. The results were expressed as the mean ± standard error (mean ± SE), with significant differences considered at a *p* < 0.05 and no significant differences considered at a *p* > 0.05.

## 3. Results

### 3.1. Growth Performance

As shown in Table 3, from days 1 to 16, group Y3 exhibited an increased ADG and BW compared to the other groups. From days 16 to 21, group L showed a decreased ADG, ADFI, and BW compared to group K, while the F/G increased. However, the ADG and BW of group Y3 were significantly higher than those of group L. Over the entire experiment (days 1 to 21), the ADG of group Y3 was significantly higher compared to group L. These findings indicate that in group Y3 the broiler chickens’ growth was promoted and the LPS-induced reduction in the ADG was reduced in a dose-dependent manner.

### 3.2. Immunoglobulin and Inflammatory Factor Content

As shown in Table 4, the LPS injection significantly increased the levels of IgM and IgG in the jejunum while significantly reducing the content of SIgA. The glutathione supplementation significantly mitigated the changes in the IgM and IgG content caused by the LPS injection and significantly increased the content of SIgA. Notably, the effect was most significant in the group receiving 200 mg/kg of reduced glutathione.

As shown in Table 5, the injection of LPS significantly increased the levels of various inflammatory factors in the jejunum, while the addition of reduced glutathione had no significant effect on IL-6 levels. However, compared with group L, the expression levels of IL-2 and TNF-α in group Y3 were significantly reduced and close to those in group K, while the expression levels of IL-4 were significantly reduced in groups Y2 and Y3, and the expression levels of IL-1β were significantly reduced in groups Y1, Y2, and Y3 (Table 5).

### 3.3. Expression Levels of TLR4/NF-κB Signaling Pathway Related Factors

**Figure 1:**
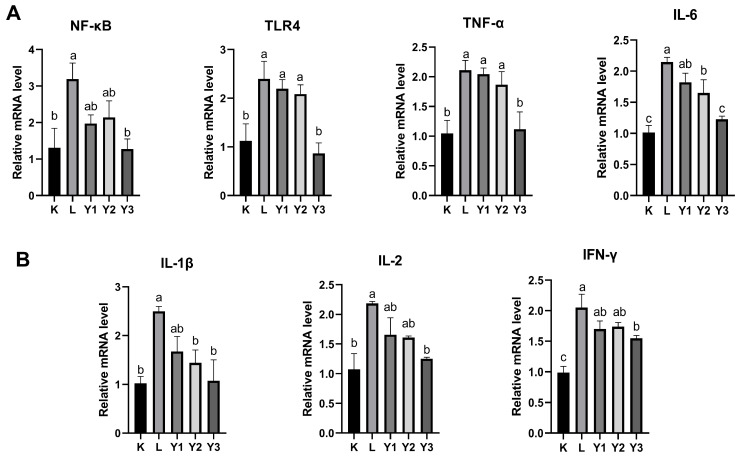
The effect of reduced glutathione on the mRNA expression level of inflammatory factors induced by LPS. (**A**) mRNA levels of *NF-κB*, *TLR4*, *TNF-α*, and *IL-6* mRNA. (**B**) mRNA levels of *IL-1β*, *IL-2*, and *IFN-γ.* n = 3. (Bars with different letters represent significant differences, *p* < 0.05).

As shown in Figure 1, LPS significantly increased the expression levels of various inflammatory factors in the K group *(p* < 0.05). Compared with the L group, the treatment in the Y2 group significantly reduced the expression levels of *IL-6* and *IL-1β* (*p* < 0.05), while the Y3 group demonstrated a more pronounced effect than the Y2 group, significantly reducing the expression levels of measured inflammatory factors (*p* < 0.05).

### 3.4. Intestinal Microbiota Community

**Figure 2:**
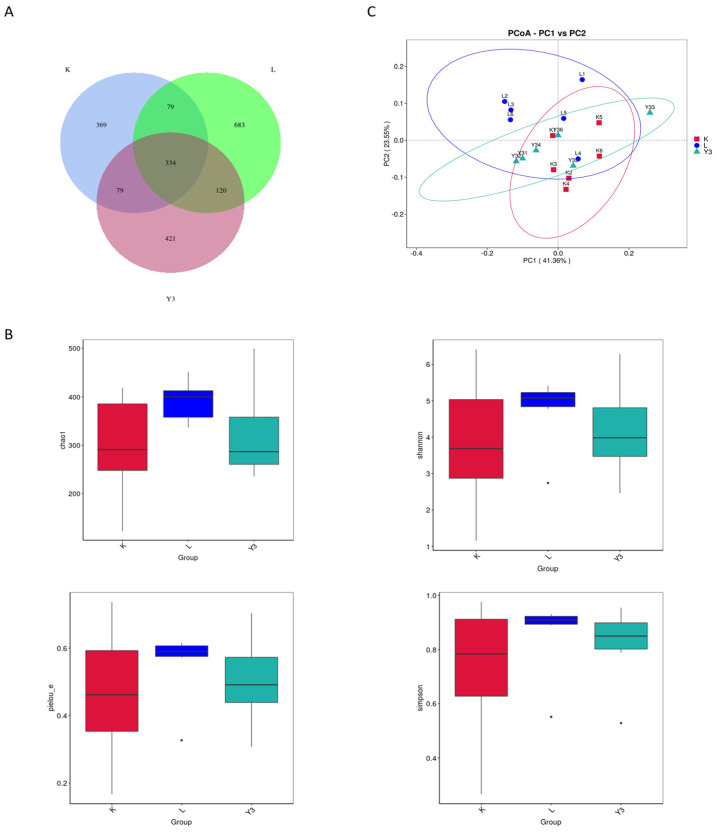
The effect of reduced glutathione on the gut microbiota of broiler chickens under an LPS attack. (**A**) Venn diagram of OTUs. (**B**) Chao1 index, Simpson index, Shannon index, and pielou_e index. (**C**) Principal Coordinate Analysis (PCoA) of gut microbiota.

**Figure 3:**
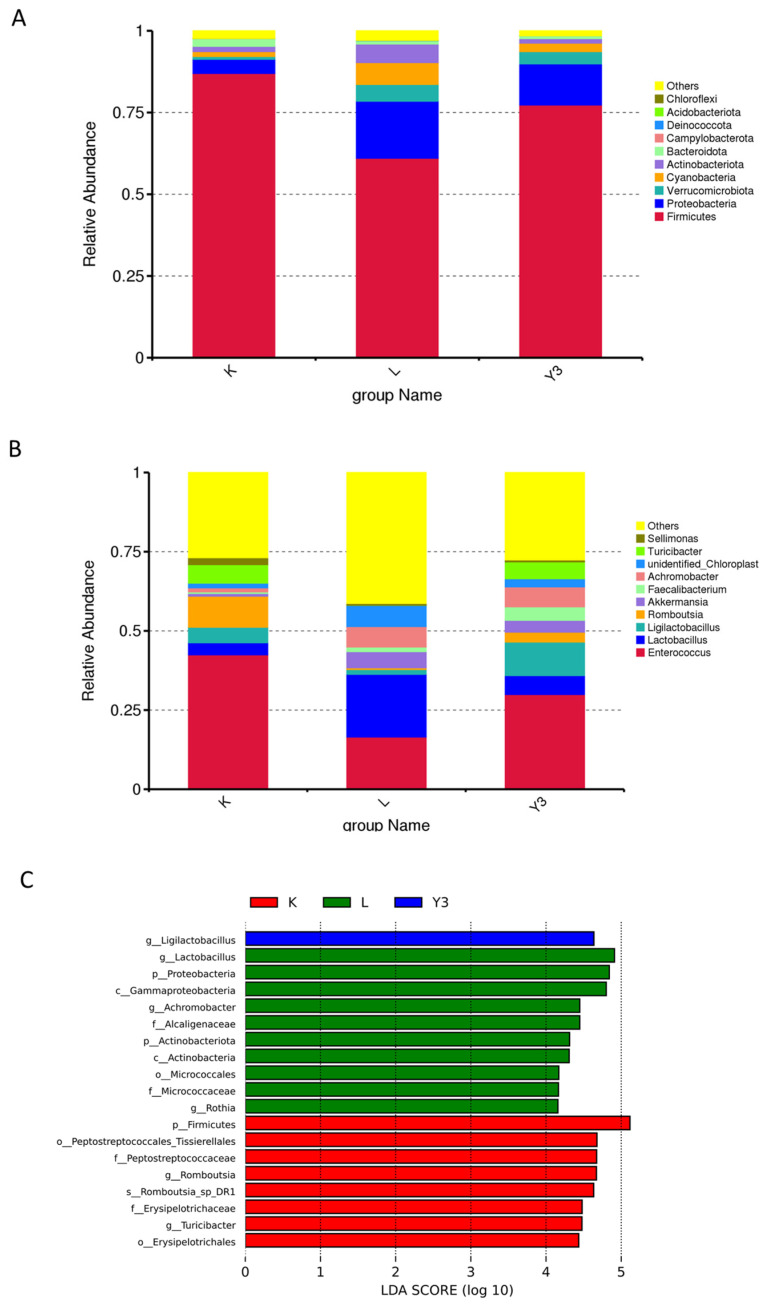
The effect of reduced glutathione on the gut microbiota of broiler chickens under an LPS attack. (**A**) The abundance of the intestinal microbiota at the phylum levels. (**B**) The abundance of the intestinal microbiota at the species levels. (**C**) LDA scores of taxonomic biomarkers identified by LEfSe (log10).

The effect of GSH on the diversity of the gut microbiota in LPS-challenged chickens is shown in Figure 2. The Venn diagram showed a total of 334 common OTUs among the three groups. The alpha diversity analysis showed no significant changes in various indices between the groups. The PCoa plot results demonstrated significant changes in the microbial community structure between the K and L groups (R = 0.47, *p* = 0.012), whereas no significant changes were observed in the microbial community structure between the K and Y3 groups (R = −0.00037, *p* = 0.415) or the L and Y3 groups (R = 0.244074, *p* = 0.057). The microbial communities across all three groups were mainly composed of *Firmicutes* and *Actinobacteria*. At the phylum level, the GSH supplementation increased the abundance of *Firmicutes* and decreased the abundance of *Proteobacteria* and *Actinobacteria* induced by the LPS challenge (Figure 3A). A further analysis at the genus level revealed that the LPS challenge significantly increased the abundance of *Enterococcus* and decreased the abundance of *Lactobacillus*, *Ligilactobacillus*, and *Turicibacter* (Figure 3B). Conversely, the GSH treatment increased the abundance of *Lactobacillus* and *Ligilactobacillus* while decreasing the abundance of *Enterococcus.* The LEfSe analysis (LDA threshold greater than four) identified *Actinobacteria* and *Proteobacteria* as characteristic taxa in the L group, *Firmicutes* in the K group, and *Ligilactobacillus* in the Y3 group (Figure 3C).

## 4. Discussion

When immune stress occurs, the body’s stress response is often more rapid than that of individual tissues and cells [24]. Under such conditions, nutritional resources originally used to promote body growth and development are allocated to the immune system to maintain its physiological function [25]. Therefore, growth performance serves as an intuitive reflection of the damage caused by stress in broiler chickens. In this study, the average daily gain of broilers was increased by the addition of 200 mg/kg of GSH from 1 to 16 days, indicating that GSH has the effect of promoting growth under normal conditions, consistent with the results of Ming et al. [26], who pointed out that adding a certain amount of GSH to the diet can promote the growth of juvenile grass carp and improve the survival rate. From days 16 to 21, 200 mg/kg of GSH significantly improved the average daily weight gain reduction caused by the LPS injection, indicating that adding 200 mg/kg of GSH can help alleviate the negative impact of immune stress on growth performance. Despite the positive achievements mentioned above, our research still has some limitations that need to be recognized. Firstly, we only conducted a 21-day study. Therefore, the long-term effects of the treatment after 21 days are still unknown, and longitudinal studies are needed in the future to determine whether the treatment has delayed effects. Secondly, we only tested the use of glutathione as a therapeutic agent in the first developmental stage (i.e., the first three weeks of life) but did not test the role of glutathione in any other developmental stage, as the physiological mechanisms that cause stress may be different from those in the first week of life. It is recommended to conduct longitudinal studies in future research to test the stage-dependent efficacy of glutathione in various environments. Finally, due to the limitations of the feeding environment, the sample size is relatively small, which may lead to low statistical power and the easy omission of small real differences. In the future, the number of replicates should be expanded, or multi-batch validation should be adopted to further confirm the accuracy of the conclusions of this study.

Immunoglobulins are critical indicators for evaluating immune status [27]. Elevated levels often indicate inflammation or a pathological state [28]. Conversely, SIgA, which mainly exists on the surface of most mucous membranes, possesses anti-inflammatory properties [29]. In this study, the SIgA content in the jejunal mucosa decreased significantly after the LPS injection, while the IgG content [30,31], which eliminates or neutralizes antigens through the complement cascade reaction and antibody-dependent cytotoxicity, significantly increased. The IgM content, which first appeared after the antigen stimulation, also significantly increased; these observations align with results reported by Lv et al. [32]. The supplementation with 200 mg/kg of GSH significantly mitigated these changes, including the reduction in the LPS-induced SIgA content surge and IgG and IgM levels. This is consistent with Zhang et al. [33], who observed that the GSH dose dependently reduces IL-4-induced IgG1 and IgG4 synthesis in specific contexts. This further indicates that GSH regulates immune factors by activating macrophages and enhancing the activity of immune cells and improving the overall immune function [27]. In addition, LPS significantly increased the levels of inflammatory factors IL-2, IL-4, IL-6, IL-1β, and TNF-α in the jejunal mucosa; similar results were observed by Tong et al. [31]. The dietary addition of 200 mg/kg of GSH effectively reduced the levels of IL-2, IL-4, IL-1β, and TNF-α, echoing the findings of Hao et al. [34], who reported that GSH significantly inhibited the TNF-α and IL-1β protein secretion in RA synovial cells. Our results indicate that the addition of 200 mg/kg of GSH has is better at reducing the levels of IL-2, IL-4, and IL-1β in the jejunum than the addition of 50 mg/kg and 100 mg/kg; furthermore, they demonstrate that 200 mg/kg of GSH alleviates the LPS-induced jejunal injury, and this dose has a better effect on injuries, and the best relief effect is achieved.

LPS activates nuclear factor kappa B in monocytes and macrophages through the signal transduction of Toll-like receptors on target cells, driving the expression of *IL-1β*, *IL-6*, and *TNF-α* [35,36]. The NF-κB pathway is involved in the gene expression of inflammatory responses and is one of the main inflammatory signaling pathways [37]. In this study, LPS significantly upregulated the relative expression levels of *NF-κB*, *TLR4*, *IL-1β*, *IL-6*, *TNF-α*, *IFN-γ*, and *IL-2* genes, similar to the results of Song et al. [38]. In contrast, the treatment with 200 mg/kg of GSH reduced the relative expression levels of *NF-κB*, *TLR4*, *IL-1β*, *IL-6*, *TNF-α*, and *IL-2*. This matches the observations in a Chinese mitten crab study, where the mRNA expressions of *TLR1* and *TLR2* in the liver and pancreas were significantly reduced when the dietary GSH level increased from 0 to 900 mg/kg [39]. Furthermore, John et al. also observed that GSH downregulates the transcription of *TNF-α*, *IL-1β*, and *IL-6* by blocking the DNA binding activity of NF-κB and AP-1 [40], which is consistent with our experimental results. Compared with the GSH addition levels of 50 mg/kg and 100 mg/kg, the GSH addition level of 200 mg/kg had a more significant effect on reducing the relative expression of *TRL4* and *TNF-α* mRNA. This suggests that the addition of 200 mg/kg of GSH to the diet maintains an optimal oxidative environment in the body, inhibiting the *TLR4/NF-κB* signaling pathway and inducing LPS-related inflammation.

The gut microbiota is a highly complex ecosystem that plays a critical role in immune and metabolic processes, as well as the overall health of the host body [41]. Existing evidence confirms that inflammatory enteritis can lead to microbial dysbiosis; while under normal conditions *Firmicutes* dominate the microbial community, *Proteobacteria* and *Actinobacteria* have a relatively low abundance [42]. However, in intestinal inflammation, the case is different because the *Proteobacteria* and *Actinobacteria* abundance is significantly increased [43]. In this study, while the alpha diversity remained stable, the β diversity analysis showed that the LPS injection significantly altered the microbial structure. The supplementation with 200 mg/kg of GSH improved these structural changes, restoring the abundance of Firmicutes while decreasing the abundance of Proteobacteria and Actinobacteria. Firmicutes produce short-chain fatty acids, such as butyric acid, which fuel intestinal epithelial cells, enhance intestinal barrier function, inhibit inflammation, and maintain immune homeostasis [44]. Thus, GSH may stabilize intestinal immune homeostasis by enhancing the production of short-chain fatty acids via the restoration of the *Firmicutes* abundance. Furthermore, the genus level analysis revealed that a concentration of 200 mg/kg of GSH increased the abundance of *Ligilactobacillus*, which promotes SCFA-producing bacteria and *Turicibacter*, which promote the differentiation of Tregs and inhibit autoimmune inflammation while decreasing the abundance of *Enterococcus* [45,46], which is known to trigger cytokine storms by releasing LTA-enriched membrane vesicles and activating *TLR2/NF-κB*. The polysaccharide chitosan facilitates the isolation of small extracellular vesicles from multiple biofluids [47]. This indicates that 200 mg/kg of GSH regulates bacterial populations that are critical for intestinal homeostasis and immune modulation, restoring microbial structural changes that are affected by the LPS injection.

## 5. Conclusions

The dietary addition of 200 mg/kg of GSH promotes the growth of broiler chickens under non-stress conditions and effectively alleviates the growth performance decline of broiler chickens under stress conditions; a GSH treatment modulates the immune status by reducing the levels of immunoglobulin and inflammatory factors in the jejunal mucosa and inhibits the relative expression levels of *TLR4/NF-κB* signaling pathway-related genes. Furthermore, GSH regulates the gut microbiota by reducing the abundance of *Enterococcus*, *Proteobacteria*, and *Actinobacteria*, while restoring *Turi-cibacter*, *Ligilactobacillus*, and *Firmicutes* to normal levels. Hence, these findings suggest that GSH is a viable nutritional intervention for maintaining intestinal health and mitigating immune stress in broilers.

## Figures and Tables

**Table 1:** The composition and nutrient levels of the basal diet.

Ingredient (%)	0~21 d (%)	Nutrient Levels	0~21 d (%)
Corn	56.60	(MJ/Kg)	11.63
Soybean meal	26.20	CP	18.0
Fish meal	4.50	CF	6.00
Limestone	1.50	Ash	8.00
Corn protein flour	3.65	Ca	1.20
Vegetable oil	2.00	AP	0.60
CaHPO_4_	1.00	Met	0.58
DL-Met (99%)	0.35	H_2_O	14.00
NaCl	0.4		
FeSO_4_	1.20		
CuSO_4_	1.10		
ZnSO_4_	0.87		
MnSO_4_	0.60		
Vitamin premix *	0.03		
Total	100		

Note: * Vitamin premix provides the following: VA 12 500 IU, VD3 2500 IU, VE 18.80 mg, VK3 2.70 mg, VB12 0.03 mg, VB2 6 mg, Biotin 0.031 mg, and phytase 0.02 mg.

**Table 2:** Primers employed in qPCR.

Gene	Accession Number	Primer Sequences (5′→3′)	bp
*β-actin*	NM_205518.2	F: GCCAACAGAGAGAAGATGACACAG R: CATCACCAGAGTCCATCACAATACC	133
*TNF-α*	NM_204267.2	F: TCAGGACAGCCTATGCCAACAAG R: TCACGATCATCTGGTTACAGGAAG	127
*IL-1β*	NM_204524.2	F: GCCGAGGAGCAGGGACTTTG R: GAAGGACTGTGAGCGGGTGTAG	136
*IL-2*	NM_204153.2	F: CTCAAGAGTCTTACGGGTCTAAATCAC R: TCTCACAAAGTTGGTCAGTTCATGG	111
*IL-6*	NM_204628.2	F: GAAATCCCTCCTCGCCAATCTG R: CCTCACGGTCTTCTCCATAAACG	106
*IFN-γ*	NM_205149.2	F: GCTGACGGTGGACCTATTATTGTAG R: GTTTGATGTGCGGCTTTGACTTG	139
*NF-κB*	NM_001396395.1	F: GCCAACAGAGAGAAGATGACACAG R: CATCACCAGAGTCCATCACAATACC	91
*TLR4*	NM_001030693.2	F: CCATCCCAACCCAACCACAGTAG R: ACCCACTGAGCAGCACCAATG	122

**Table 3:** The effect of reduced glutathione on the growth performance of broiler chickens.

Stage	Item	K	L	Y1	Y2	Y3	SEM	*p* Value
1–16 d	BW/g	43.04	41.61	41.54	42.90	42.36	0.28	0.257
1–16 d	ADG/g	22.56 ^bc^	21.00 ^c^	22.83 ^bc^	23.34 ^ab^	24.04 ^a^	0.14	<0.001
1–16 d	ADFI/g	41.76	40.92	41.07	39.74	41.17	2.47	1
1–16 d	BW/g	381.45 ^bc^	377.30 ^c^	384.02 ^bc^	392.95 ^ab^	403.27 ^a^	2.07	<0.001
1–16 d	F/G	1.88	1.86	1.72	1.73	1.69	0.12	0.956
16–21 d	ADG/g	34.93 ^a^	22.51 ^c^	23.32 ^c^	24.01 ^bc^	28.93 ^b^	0.89	<0.001
16–21 d	ADFI/g	94.50 ^b^	81.56 ^a^	84.97 ^a^	86.92 ^ab^	91.40 ^ab^	1.43	0.049
16–21 d	BW/g	588.65 ^a^	498.75 ^d^	523.15 ^cd^	542.30 ^bc^	576.40 ^ab^	5.44	<0.001
16–21 d	F/G	2.76 ^b^	3.89 ^a^	3.56 ^ab^	3.66 ^ab^	3.25 ^ab^	0.3	0.027
1–21 d	ADG/g	25.99 ^ab^	21.76 ^a^	22.88 ^ab^	23.80 ^bc^	25.40 ^c^	0.26	<0.001
1–21 d	ADFI/g	56.84	52.53	53.61	53.22	55.52	2.78	0.988
1–21 d	F/G	2.24	2.48	2.40	2.30	2.20	0.12	0.877

Group K received the basic feed; group L received the basic feed; group Y1 received the basic feed supplemented with 50 mg/kg of reduced glutathione; group Y2 received the basic feed supplemented with 100 mg/kg of reduced glutathione; and group Y3 received the basic feed supplemented with 200 mg/kg of reduced glutathione. Results are presented as the mean and standard error of the mean (SEM). For the 1–16 day BW, ADG (n = 44), ADFI (n = 21), and F/G (n = 20). For the 16–21 day ADG (n = 20), ADFI (n= 21), and F/G (n = 20). For the 1–21 day ADG (n= 20), ADFI (n= 21), and F/G (n = 20). Within the same row, values sharing the same letter indicate no significant difference (*p* > 0.05), while groups without the same letters indicate a significant difference (*p* < 0.05).

**Table 4:** The effect of reduced glutathione on intestinal immunoglobulin attacked by LPS.

Items	Groups					SEM	*p* Value
	K	L	Y1	Y2	Y3		
sIgA (μg/mL)	14.00 ^b^	11.62 ^c^	14.90 ^ab^	14.13 ^b^	16.10 ^a^	0.201	<0.001
IgM (μg/mL)	540.46 ^c^	802 ^a^	729.98 ^b^	726.99 ^b^	590.67 ^c^	9.522	<0.001
IgG (μg/mL)	2245.31 ^d^	2584.38 ^a^	2468.75 ^b^	2315.63 ^c^	2310.16 ^c^	3.29	<0.001

Group K: basic feed; group L: basic feed; group Y1: basic feed supplemented with 50 mg/kg of reduced glutathione; group Y2: basic feed supplemented with 100 mg/kg of reduced glutathione; and group Y3: basic feed supplemented with 200 mg/kg of reduced glutathione. Results are presented as the mean and standard error of the mean (SEM);. For sIgA and IgG, n = 4; IgM, n = 3. Within the same row, values sharing the same letter indicate no significant difference (*p* > 0.05), while those with different letters indicate a significant difference (*p* < 0.05).

**Table 5:** The effect of reduced glutathione on intestinal inflammatory factors attacked by LPS.

Items	Groups					SEM	*p* Value
	K	L	Y1	Y2	Y3		
IL-2 (p g/mL)	226.50 ^b^	285.65 ^a^	269.31 ^a^	267.35 ^a^	231.73 ^b^	4.387	0.006
IL-6 (p g/mL)	26.4 ^a^	34.65 ^b^	31.25 ^ab^	30.84 ^ab^	30.24 ^ab^	0.669	0.024
IL-4 (p g/mL)	11.06 ^c^	12.61 ^a^	12.42 ^ab^	12.07 ^b^	10.62 ^d^	0.055	<0.001
IL-1β (p g/mL)	909.48 ^d^	1340.52 ^a^	1246.55 ^b^	1018.53 ^c^	981.90 ^c^	7.17	<0.001
TNF-α (p g/mL)	113.74 ^c^	128.34 ^a^	120.75 ^ab^	123.79 ^ab^	107.10 ^c^	1.835	0.018

Group K: basic feed; group L: basic feed; group Y1: basic feed supplemented with 50 mg/kg of reduced glutathione; group Y2: basic feed supplemented with 100 mg/kg of reduced glutathione; and group Y3: basic feed supplemented with 200 mg/kg of reduced glutathione t. Results are presented as the mean and standard error of the mean (SEM). IL-2, IL-6, n = 3; IL-4, IL-1β, TNF-α, n = 4. Within the same row, values with the same letter indicate no significant difference (*p* > 0.05), while values with different letters indicate a significant difference (*p* < 0.05).

## Data Availability

The data in the study can be obtained from the corresponding author.

## Abbreviations

The following abbreviations are used in this manuscript:

GSH	glutathione
LPS	lipopolysaccharide
ADG	average daily gain
ADFI	average daily feed intake
F/G	average daily feed intake/average daily gain
NF-κB	nuclear factor kappa-B
TLR4	toll-like receptor 4

## References

[1] Liu H., Meng H., Du M., Lv H., Wang Y., Zhang K. (2024). Chlorogenic acid ameliorates intestinal inflammation by inhibiting NF-κB and endoplasmic reticulum stress in lipopolysaccharide-challenged broilers. Poult. Sci..

[2] Oliphant K., Allen-Vercoe E. (2019). Macronutrient metabolism by the human gut microbiome: Major fermentation by-products and their impact on host health. Microbiome.

[3] Chen L., Deng H., Cui H., Fang J., Zhao L. (2015). Inflammatory responses and inflammation-associated diseases in organs. Oncotarget.

[4] Wang X., Zhu L., Li X., Wang X., Hao R., Li J. (2022). Effects of high fructose corn syrup on intestinal microbiota structure and obesity in mice. npj Sci. Food.

[5] Diana A., Manzanilla E.G., Díaz J.A.C., Leonard F.C., A Boyle L. (2017). Do weaner pigs need in-feed antibiotics to ensure good health and welfare?. PLoS ONE.

[6] Zielen S., Trischler J., Schubert R. (2015). Lipopolysaccharide challenge: Immunological effects and safety in humans. Expert Rev. Clin. Immunol..

[7] Mireles A.J., Kim S.M., Klasing K.C. (2005). An acute inflammatory response alters bone homeostasis, body composition, and the humoral immune response of broiler chickens. Poult. Sci..

[8] Yang X.J., Li W.L., Feng Y., Yao J.H. (2011). Effects of immune stress on growth performance, immunity, and cecal microflora in chickens. Poult. Sci..

[9] Duan T., Du Y., Xing C., Wang H.Y., Wang R.F. (2022). Toll-Like Receptor Signaling and Its Role in Cell-Mediated Immunity. Front. Immunol..

[10] Hong Y., Lee J., Vu T.H., Lee S., Lillehoj H.S., Hong Y.H. (2021). Exosomes of lipopolysaccharide-stimulated chicken macrophages modulate immune response through the MyD88/NF-κB signaling pathway. Dev. Comp. Immunol..

[11] Zhang C., Li C., Shao Q., Chen W., Ma L., Xu W., Li Y., Huang S., Ma Y. (2021). Effects of Glycyrrhiza polysaccharide in diet on growth performance, serum antioxidant capacity, and biochemistry of broilers. Poult. Sci..

[12] Dringen R., Pfeiffer B., Hamprecht B. (1999). Synthesis of the antioxidant glutathione in neurons: Supply by astrocytes of CysGly as precursor for neuronal glutathione. J. Neurosci..

[13] Liu S.M., Eady S.J. (2005). Glutathione: Its implications for animal health, meat quality, and health benefits of consumers. Aust. J. Agric. Res..

[14] Owen J.B., Butterfield D.A. (2010). Measurement of oxidized/reduced glutathione ratio. Protein Misfolding and Cellular Stress in Disease and Aging; Methods in Molecular Biology.

[15] Schmitt B., Vicenzi M., Garrel C., Denis F.M. (2015). Effects of N-acetylcysteine, oral glutathione (GSH) and a novel sublingual form of GSH on oxidative stress markers: A comparative crossover study. Redox Biol..

[16] Qin J., Wang C., Zhou X. (2024). Glutathione regulates CIA-activated splenic lymphocytes via NF-κB/MMP-9 and MAPK/PCNA pathways manipulating immune response. Cell Immunol..

[17] Chen Y., Chen M., Zhai T., Zhou H., Zhou Z., Liu X., Yang S., Yang H. (2022). Glutathione-responsive chemodynamic therapy of manganese (III/IV) cluster nanoparticles enhanced by electrochemical stimulation via oxidative stress pathway. Bioconjug. Chem..

[18] Pastore A., Federici G., Bertini E., Piemonte F. (2003). Analysis of glutathione: Implication in redox and detoxification. Clin. Chim. Acta.

[19] Fang Y.Z., Yang S., Wu G. (2002). Free radicals, antioxidants, and nutrition. Nutrition.

[20] Xue S., Chen S., Ge Y., Guan T., Han Y. (2022). Regulation of glutathione on growth performance, biochemical parameters, non-specific immunity, and related genes of common carp (*Cyprinus carpio*) exposed to ammonia. Aquaculture.

[21] Li Y., Zhang H., Chen Y., Yang M., Zhang L., Lu Z., Zhou Y.M., Wang T. (2015). *Bacillus amyloliquefaciens* supplementation alleviates immunological stress in lipopolysaccharide-challenged broilers at early age. Poult. Sci..

[22] Magoc T., Salzberg S.L. (2011). FLASH: Fast length adjustment of short reads to improve genome assemblies. Bioinformatics.

[23] Edgar R.C., Haas B.J., Clemente J.C., Quince C., Knight R. (2011). UCHIME improves sensitivity and speed of chimera detection. Bioinformatics.

[24] Kassahn K.S., Crozier R.H., Prtner H.O., Caley M.J. (2010). Animal performance and stress: Responses and tolerance limits at different levels of biological organisation. Biol. Rev..

[25] De Rosa V., Galgani M., Santopaolo M., Colamatteo A., Laccetti R., Matarese G. (2015). Nutritional control of immunity: Balancing the metabolic requirements with an appropriate immune function. Semin. Immunol..

[26] Ming J.H., Ye J.Y., Zhang Y.X., Xu P., Xie J. (2015). Effects of dietary reduced glutathione on growth performance, non-specific immunity, antioxidant capacity and expression levels of IGF-I and HSP70 mRNA of grass carp (*Ctenopharyngodon idella*). Aquaculture.

[27] Liao X.D., Ma G., Cai J., Fu Y., Yan X.Y., Wei X.B., Zhang R.J. (2015). Effects of Clostridium butyricum on growth performance, antioxidation, and immune function of broilers. Poult. Sci..

[28] Tan T.T., Coussens L.M. (2007). Humoral immunity, inflammation and cancer. Curr. Opin. Immunol..

[29] Hooper L.V., Alt F.W. (2015). Epithelial cell contributions to intestinal immunity. Advances in Immunology.

[30] Schroeder H.W., Cavacini L. (2010). Structure and function of immunoglobulins. J. Allergy Clin. Immunol..

[31] Tong X., Zhang J., Li J. (2020). LPS-induced inflammation disorders bone modeling and remodeling by inhibiting angiogenesis and disordering osteogenesis in chickens. Inflamm. Res..

[32] Lv H., Li P., Wang Z., Gao M., Li G., Nie W., Xiao L., Lv Z., Guo Y. (2023). Effects of Dietary Supplemental Chlorogenic Acid and Baicalin on the Growth Performance and Immunity of Broilers Challenged with Lipopolysaccharide. Life.

[33] Zhang Z., Zhang X., Fang X., Niimi M., Huang Y., Piao H., Gao S., Fan J., Yao J. (2017). Glutathione inhibits antibody and complement-mediated immunologic cell injury via multiple mechanisms. Redox Biol..

[34] Hao W.T., Huang L., Pan W., Ren Y.L. (2022). Antioxidant glutathione inhibits inflammation in synovial fibroblasts via PTEN/PI3K/AKT pathway: An in vitro study. Arch. Rheumatol..

[35] Cao S.G., Chen R., Wang H., Lin L.M., Xia X.P. (2018). Cryptotanshinone inhibits prostaglandin E2 production and COX-2 expression via suppression of TLR4/NF-κB signaling pathway in LPS-stimulated Caco-2 cells. Microb. Pathog..

[36] Stephens M., von der Weid P.Y. (2020). Lipopolysaccharides modulate intestinal epithelial permeability and inflammation in a species-specific manner. Gut Microbes.

[37] Ilchovska D., Barrow D.M. (2021). An overview of the NF-κB mechanism of pathophysiology in rheumatoid arthritis, investigation of the NF-κB ligand RANKL and related nutritional interventions. Autoimmun. Rev..

[38] Song Z., Zhao T., Liu L., Jiao H., Lin H. (2011). Effect of copper on antioxidant ability and nutrient metabolism in broiler chickens stimulated by lipopolysaccharides. Arch. Anim. Nutr..

[39] Liu J.D., Liu W.B., Zhang D.D., Xu C.Y., Zhang C.Y., Zheng X.C., Chi C. (2020). Dietary reduced glutathione supplementation can improve growth, antioxidant capacity, and immunity on Chinese mitten crab, *Eriocheir sinensis*. Fish Shellfish Immunol..

[40] Haddad J.J., Harb H.L. (2005). L-γ-Glutamyl-L-cysteinyl-glycine (glutathione; GSH) and GSH-related enzymes in the regulation of pro- and anti-inflammatory cytokines: A signaling transcriptional scenario for redox(y) immunologic sensor(s)?. Mol. Immunol..

[41] Portincasa P., Bonfrate L., Vacca M., De Angelis M., Farella I., Lanza E., Khalil M., Wang D.Q.-H., Sperandio M., Di Ciaula A. (2022). Gut Microbiota and Short Chain Fatty Acids: Implications in Glucose Homeostasis. Int. J. Mol. Sci..

[42] Miyoshi J., Chang E.B. (2017). The gut microbiota and inflammatory bowel diseases. Transl. Res..

[43] Mukhopadhya I., Hansen R., El-Omar E.M., Hold G.L. (2012). IBD-what role do Proteobacteria play?. Nat. Rev. Gastroenterol. Hepatol..

[44] Wang F., Wang X., Wang C., Yan W., Xu J., Song Z., Su M., Zeng J., Han Q., Ruan G. (2025). Gut microbiota-derived glutathione from metformin treatment alleviates intestinal ferroptosis induced by ischemia/reperfusion. BMC Med..

[45] Meimandipour A., Shuhaimi M., Soleimani A.F., Azhar K., Hair-Bejo M., Kabeir B.M., Javanmard A., Anas O.M., Yazid A.M. (2010). Selected microbial groups and short-chain fatty acids profile in a simulated chicken cecum supplemented with two strains of *Lactobacillus*. Poult. Sci..

[46] Hamada K., Isobe J., Hattori K., Hosonuma M., Baba Y., Murayama M., Narikawa Y., Toyoda H., Funayama E., Tajima K. (2023). *Turicibacter* and *Acidaminococcus* predict immune-related adverse events and efficacy of immune checkpoint inhibitor. Front. Immunol..

[47] Kumar A., Dhadi S.R., Mai N.N., Taylor C., Roy J.W., Barnett D.A., Lewis S.M., Ghosh A., Ouellette R.J. (2021). The polysaccharide chitosan facilitates the isolation of small extracellular vesicles from multiple biofluids. J. Extracell. Vesicles.

