# Correction: Wang et al. The Effects of Reduced Glutathione on Growth Performance, Intestinal Inflammation, and Gut Microbiota in Immune-Stressed Broiler Chickens. *Animals* 2026, *16*, 178

**DOI:** 10.3390/ani16091359

**Published:** 2026-04-29

**Authors:** Xin-Qi Wang, Tao Zhang, Ying-Kun Liu, Hao-Jia Li, Kabelo Anthony Makatjane, Zhen Lai, Jian-Xin Bi, Hai-Zhu Zhou, Wei Guo

**Affiliations:** 1 College of Animal Science and Technology, Jilin Agricultural University, Changchun 130118, China; 18943857722@163.com (X.-Q.W.); 18188114727@163.com (Y.-K.L.); 18932783018@163.com (H.-J.L.); anthonymakatjane@gmail.com (K.A.M.); 13147728730@163.com (Z.L.); 13251763422@163.com (J.-X.B.); 2 Jilin Provincial Science and Technology Innovation Platform Management Center, Changchun 130012, China; anbigboy@126.com; 3 Jilin Provincial Livestock Station, Changchun 130000, China

## Additional Affiliations

In the published paper [1], there was an error regarding the affiliations of Tao Zhang and Wei Guo. The affiliation of Tao Zhang should be changed to Jilin Provincial Science and Technology Innovation Platform Management Center, and that of Wei Guo to Jilin Provincial Livestock Station. The author contributions should be noted with † for the first two authors who made equal contributions. The authors state that the scientific conclusions are unaffected. This correction was approved by the Academic Editor. The original publication has also been updated.

## References

[1] Wang X.-Q., Zhang T., Liu Y.-K., Li H.-J., Makatjane K.A., Lai Z., Bi J.-X., Zhou H.-Z., Guo W. (2026). The Effects of Reduced Glutathione on Growth Performance, Intestinal Inflammation, and Gut Microbiota in Immune-Stressed Broiler Chickens. Animals.

